# Dried Blood Spot Whole-Blood Fatty-Acid Profiles and Maximum Carotid Wall Thickness in Chinese Adults with Carotid Structural Involvement: A Dual-Route Compositional Cross-Sectional Study

**DOI:** 10.3390/nu18213495

**Published:** 2026-10-24

**Authors:** Yetong Ma, Mengke Zhang, Xin Qin, Xinsheng Zhang, Lu Liu, Yinghua Liu

**Affiliations:** 1 Department of Nutrition, First Medical Center, Chinese PLA General Hospital, Beijing 100853, China; mytimoney@163.com (Y.M.);; 2 Graduate School of The PLA General Hospital, Beijing 100853, China; 3 Department of Ultrasound, First Medical Center, Chinese PLA General Hospital, Beijing 100853, China

**Keywords:** dried blood spot, whole-blood fatty acids, compositional data analysis, isometric log-ratio, maximum carotid wall thickness, nutritional–metabolic phenotyping, cardiovascular disease prevention

## Abstract

**Background/Objectives:** Questionnaires regarding intake and routine lipid panels do not indicate fatty-acid composition. Dried blood spot (DBS) whole-blood profiling is a minimally invasive complement not yet linked to a vascular structural endpoint. We tested whether DBS composition tracks carotid wall burden in Chinese adults with metabolic-risk features and established carotid structural involvement using a prespecified panel and compositional analysis. **Methods:** Fasting DBS gas chromatography resolved 37 fatty acids in 230 adults (47.4% men) meeting a prespecified maximum carotid wall thickness (max-CWT) ≥ 1.0 mm eligibility criterion. A total of 16 prespecified measures were modelled against ln(max-CWT) with multivariable adjustment and Benjamini–Hochberg correction pooled across 16 tests; the 26-part composition was examined by isometric log-ratio (ILR) analysis. **Results:** Under pooled false-discovery-rate control, α-linolenic acid (ALA) and linoleic acid (LA) were inversely and oleic acid positively associated with max-CWT (per 1-SD β = −0.063, −0.068 and 0.080; all q < 0.05). The full composition was associated (F(25,196) = 2.26, *p* = 0.0011), whereas EPA and the AA/EPA ratio remained weaker, family-level signals. **Conclusions:** To our knowledge, this is the first dual-route DBS compositional analysis linked to a structural vascular endpoint, and it identified a candidate nutritional–metabolic pattern—lower ALA and LA and higher oleic acid—linked to wall thickness in adults with established structural involvement. This pattern warrants prospective investigation and does not by itself support any dietary recommendation.

## 1. Introduction

Accurate nutritional assessment is central to preventing and managing cardiometabolic disease, yet the two instruments used most often each capture only part of the picture. Dietary questionnaires record what people report eating and are vulnerable to recall and reporting bias, whereas routine lipid biochemistry reports aggregate lipoprotein fraction measures—total, LDL- and HDL–cholesterol and circulating triglycerides—but not the fatty-acid composition of these lipid pools [1,2,3,4,5,6]. An objective, minimally invasive and repeatable measure of internal fatty-acid status would therefore fill a practical gap in clinical nutrition. Dried blood spot (DBS) whole-blood profiling meets this need: a fingertip sample integrates dietary absorption with endogenous metabolism, includes both plasma and cellular lipid fractions, and can be collected serially to monitor nutritional intervention [7,8,9,10,11].

Carotid wall burden is a well-validated marker of systemic atherosclerosis and predicts recurrent stroke and cardiovascular events beyond conventional risk factors [12,13,14,15]. Fatty acids are plausible nutritional determinants of this process. Long-chain *n*-3 polyunsaturated fatty acids (PUFAs) enter membrane phospholipids and shape oxylipin and inflammatory pathways, but randomised trials of marine supplementation have reached mixed conclusions [16,17,18,19,20]. The essential plant-derived PUFAs α-linolenic acid (ALA) and linoleic acid (LA) have shown inverse associations with cardiovascular events and mortality in prospective cohorts [21,22,23,24,25], while monounsaturated fatty acids and the balance between *n*-6 and *n*-3 pools remain debated [26,27,28]. Most biomarker evidence, however, comes from plasma or erythrocyte membranes in Western populations and analyses one fatty acid at a time [29,30,31,32,33,34,35].

A methodological issue compounds this gap. Because fatty-acid percentages sum to a fixed total, they form a closed composition: an increase in one component necessarily lowers the relative contribution of the others. Conventional measure-by-measure regression ignores this constraint, whereas log-ratio methods explicitly model relative composition and avoid spurious interpretation [36,37]. We are not aware of a previous study that has applied composition-aware analysis to DBS whole-blood profiles linked to a vascular structural outcome: published DBS fatty-acid work established assay feasibility, reproducibility, dietary responsiveness and population-level status monitoring but was not paired with structural vascular imaging, whereas carotid biomarker studies relied on erythrocyte membrane, serum, plasma or venous whole blood rather than DBS. We are likewise not aware of a study that ran a prespecified conventional panel and a closed-composition analysis in parallel within one cohort so that the two routes could check one another.

Chinese adults with metabolic-risk features are an informative setting in which to address these questions: their diets and edible-oil patterns differ from olive oil-rich Western diets, and an *n*-6-dominant fatty-acid profile is common [27,28]. To our knowledge, no previous study has combined a low-volume DBS whole-blood assay with carotid ultrasonography in adults with carotid structural involvement; we did so in 230 Chinese adults, deliberately pairing two independently parameterised analytic strategies so that each could cross-check the other. This study had three prespecified objectives: (i) to characterise the DBS fatty-acid profile as an objective nutritional–metabolic phenotype that complements questionnaires and routine biochemistry; (ii) to relate a prespecified 16-measure panel and the full closed composition to maximum carotid wall thickness (max-CWT), using two complementary analytic routes for internal concordance and published prospective cohorts for contextual corroboration; and (iii) to identify a candidate fatty-acid pattern associated with wall burden that could be tested in future longitudinal and, where warranted, dietary intervention studies with repeated DBS sampling.

## 2. Materials and Methods

### 2.1. Study Design, Setting, and Ethics

This single-centre cross-sectional study was conducted at the First Medical Center, Chinese PLA General Hospital, between December 2025 and August 2026. It was approved by the Medical Ethics Committee of the Chinese PLA General Hospital (No. S2025-647-02; approved 18 September 2025) and prospectively registered in the Chinese Clinical Trial Registry (ChiCTR2500113737; registered 2 December 2025) before recruitment began. Every participant provided written informed consent before any study procedure, and the study followed the Declaration of Helsinki. The reporting adheres to the STROBE statement for cross-sectional studies.

### 2.2. Participants and Eligibility

**Figure 1:**
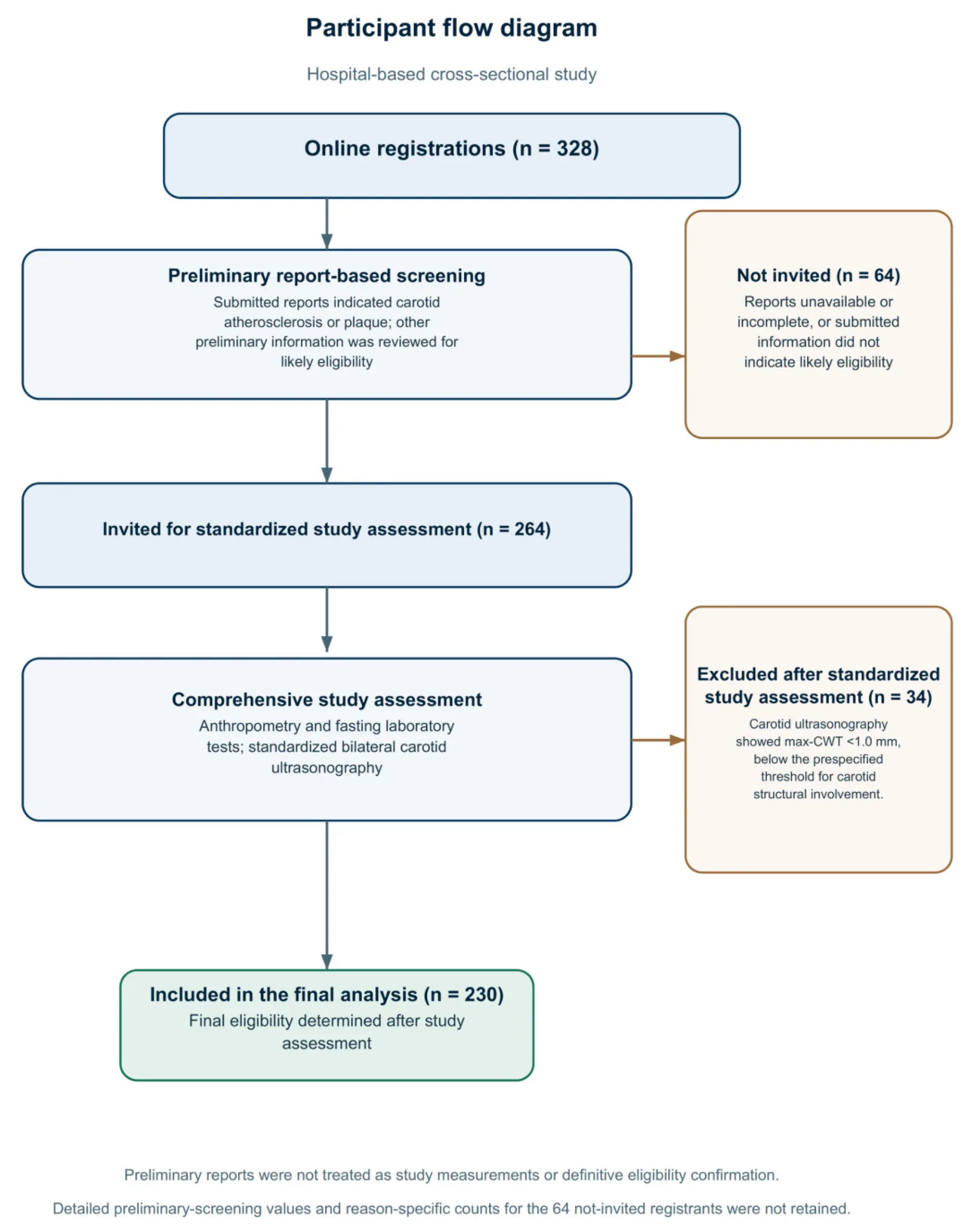
Participant flow from registration to the final analytic sample. Of 328 registered individuals, 64 were not invited because the submitted information was unavailable or incomplete; the remaining 264 completed standardised anthropometry, fasting laboratory testing, and bilateral carotid ultrasonography, and 34 were then excluded because max-CWT was below 1.0 mm, leaving 230 participants with study-defined carotid structural involvement.

Adults were recruited through the hospital’s official online platform and screened at the nutrition and carotid ultrasound services for metabolic-risk features. Recruitment used consecutive clinical enrolment at a single centre. Eligibility required ultrasound-defined max-CWT of at least 1.0 mm plus at least one objective metabolic-risk criterion: waist-to-hip ratio (WHR) of at least 0.90 in men or 0.85 in women; fasting triglycerides of at least 1.7 mmol/L; LDL-cholesterol (LDL-C) of at least 3.4 mmol/L; HDL cholesterol (HDL-C) below 1.0 mmol/L in men or 1.3 mmol/L in women; or fasting plasma glucose (FPG) of at least 6.1 mmol/L. Exclusion criteria were an acute cardiovascular or cerebrovascular event within the preceding three months; severe hepatic, renal or haematological disease; active malignant disease; active HIV, hepatitis C or hepatitis B infection; pregnancy or lactation; and missing key clinical or laboratory data. Recent clinical reports voluntarily supplied at registration were used only for preliminary feasibility screening to invite likely-eligible individuals for the standardised assessment and were neither study measurements nor definitive eligibility determinations. Of 328 individuals who registered, 64 were not invited because their submitted information was unavailable or incomplete and therefore contributed neither exposure nor outcome data; the remaining 264 underwent standardised anthropometry, fasting laboratory testing and bilateral carotid ultrasonography, and 34 were then excluded because max-CWT was below 1.0 mm, leaving 230 participants with study-defined carotid structural involvement for analysis (Figure 1). Eligibility thus defined the clinically relevant target population (adults with metabolic-risk features and carotid structural involvement for whom clinical–nutrition assessment and intervention are clinically indicated), and the analysis addressed within-population gradients in wall burden rather than case–control contrasts. All 230 participants had complete primary-model data, and no imputation was performed; the analytic set was therefore a complete-case set, whereas variable-level missingness among individuals screened before these exclusions was not separately archived.

Sample size was calculated for the primary exposure-specific multivariable linear regression. Each Model 3 contained one standardised fatty-acid exposure together with the eight covariates (nine predictors in total). At a two-sided α of 0.05 and 80% power, a noncentral-F test of the single exposure coefficient with one numerator degree of freedom under Cohen’s f^2^ = 0.10 required 81 analysable participants; allowing for 20% attrition during screening and assessment yielded a target of 102, which the final analytic sample of 230 exceeded. This calculation concerns the prespecified single-exposure primary regressions; the 26-part compositional model was planned as exploratory and did not define the sample size, and its adequacy and degree of overfitting were examined after analysis using residual degrees of freedom, cross-validation, and a permutation test.

### 2.3. DBS Collection and Fatty-Acid Measurement

Fasting fingertip capillary whole blood was applied directly to DBS cards, dried at room temperature, and transported sealed under cold chain to the testing laboratory (Omega Bandz Biotechnology (Shanghai) Co., Ltd., Shanghai, China). One punched disc per card underwent direct transmethylation with 14% boron trifluoride in methanol, toluene, and methanol (35:30:35, *v*/*v*/*v*), followed by shaking and heating at 100 °C for 45 min. After cooling, equal volumes of hexane and distilled water were added; the upper fatty-acid methyl ester (FAME) hexane layer was analysed on an Agilent 7820 gas chromatograph (Agilent Technologies, Santa Clara, CA, USA) with a DB-23 60 m column and flame ionisation detection. Species were identified by retention time against a commercial FAME reference mixture injected with every batch. In total, 37 species were identified from the laboratory’s predefined chromatographic peak list. Measures are expressed as percentages of total identified fatty-acid peak area and therefore convey relative, not absolute, composition. Because no paired plasma or erythrocyte sample was collected, analytes are defined strictly as DBS whole-blood fatty-acid measures, which are not interchangeable with plasma or membrane values. Analyses followed the testing laboratory’s standardised GC-FID workflow under its routine quality control programme, with the commercial FAME reference mixture run in every batch to anchor retention time identification and run-to-run consistency. Analytical performance of this DBS GC-FID workflow is supported by the laboratory’s standard operating procedures and by the published DBS methodology documenting assay correlation, storage stability, and reproducibility [7,38,39,40,41,42,43].

The prespecified 16-measure panel comprised total *n*-3 PUFA, total *n*-6 PUFA, total monounsaturated fatty acids (MUFA), total saturated fatty acids (SFA), the arachidonic-acid/eicosapentaenoic-acid (AA/EPA) ratio, the *n*-6/*n*-3 ratio, the study-defined *n*-3 index (EPA + docosahexaenoic acid (DHA)), ALA (18:3n-3), EPA (20:5n-3), DHA (22:6n-3), LA (18:2n-6), arachidonic acid (20:4n-6), adrenic acid (22:4n-6), oleic acid (18:1n-9), nervonic acid (24:1n-9) and palmitic acid (16:0); class totals were obtained by summing the corresponding percentages. For exploratory pathway analyses, SCD1-16 was calculated as cis-16:1n-7/16:0 and SCD1-18 as cis-18:1n-9/18:0; these derived ratios are compositional proxies rather than direct enzyme activity measures.

For the composition-aware analysis, the 12 species detected in fewer than 95% of participants (10:0, 11:0, 12:0, 13:0, 14:0, 14:1, 15:1, 18:1n-9t, 18:3n-6, 21:0, 20:3n-3 and 22:2n-6) were pooled into a single other_FA part, leaving 25 retained species plus other_FA (a 26-part composition; Supplementary Table S1). Zero values were replaced by k times the component-specific minimum positive value (primary k = 0.5; sensitivity k = 0.1 and 1.0), after which each composition was closed to sum to 1 [44].

### 2.4. Carotid Ultrasonography and Outcomes

Bilateral carotid ultrasonography was performed by one certified vascular sonographer using a Philips EPIQ CVx system (Philips Medical Systems, Bothell, WA, USA) fitted with a 3–12 MHz linear-array transducer; the sonographer was blinded to all fatty-acid and clinical laboratory results. Participants were examined supine, with the head rotated about 45° away from the scanned side, and the common carotid artery, carotid bifurcation and proximal internal and external carotid arteries were imaged bilaterally according to the 2025 Shanghai expert consensus [45]. Carotid plaque was defined as a focal structure meeting at least one of the following: thickness of at least 1.5 mm, protrusion into the lumen by at least 0.5 mm, or thickness exceeding the adjacent intima–media thickness (IMT) by more than 50% [45]. The primary outcome, max-CWT, was the greatest wall thickness across all bilateral scanned sites, encompassing both increased IMT and focal plaque; in participants with several plaques, the thickest lesion was designated the index lesion. Max-CWT is therefore a continuous composite structural measure, not conventional mean IMT and not a discrete plaque-burden or plaque-area endpoint; it was chosen to summarise overall wall burden on a continuous scale, to avoid the information loss from dichotomisation, and because a single maximum read with high repeatability could be obtained reliably. Per-segment labels separating plaque-bearing from purely IMT-thickened sites were not retained, so the two phenotypes could not be modelled separately. At the most stenotic site, the residual lumen diameter (R) and the estimated original lumen diameter (D2) were recorded, and percentage diameter stenosis was calculated by the European Carotid Surgery Trial (ECST) method as (1 − R/D2) × 100% [45]. For descriptive and ordered-outcome analyses, max-CWT was categorised as mild (≤1.5 mm), moderate (>1.5 to <2.5 mm) or severe (≥2.5 mm); the ≥1.0 mm inclusion threshold was an operational eligibility cut-off rather than an externally validated plaque threshold.

Intra-observer reproducibility was assessed in 78 participants randomly selected from the analytic cohort: the same reader re-analysed stored Digital Imaging and Communications in Medicine (DICOM) images at least four weeks after the initial reading, without access to the earlier measurements or to participant characteristics, and 74 participants had valid paired stenosis readings. Agreement was quantified with the two-way random-effects, absolute-agreement intraclass correlation coefficient ICC(2,1), with 95% confidence intervals obtained by percentile bootstrap; the relative standard deviation of paired differences, Bland–Altman 95% limits of agreement, repeatability coefficients, and paired t tests for systematic bias were also computed.

### 2.5. Covariates

Trained staff recorded age and sex with a standardised questionnaire; body mass index (BMI) and WHR were measured by certified personnel with a calibrated H-key350 body composition analyser (Sihai Huachen Biotechnology, Beijing, China) under standard operating procedures. After an overnight fast of at least 12 h, 5 mL of venous blood was drawn from the antecubital vein, centrifuged at 3000× *g* for 10 min and transported on a 4 °C cold chain to Adicon Clinical Laboratories (Beijing, China), an ISO 15189-accredited laboratory, within 2 h. FPG, alanine aminotransferase, serum creatinine, uric acid, triglycerides, HDL-C, LDL-C, apolipoprotein A-I (ApoA-I) and apolipoprotein B (ApoB) were assayed by automated enzymatic or immunoturbidimetric methods under routine internal and external quality control; the estimated glomerular filtration rate (eGFR) was derived with the Chronic Kidney Disease Epidemiology Collaboration (CKD-EPI) 2021 creatinine equation without a race coefficient [46]. The triglyceride–glucose index (TyG) was ln[triglycerides (mg/dL) × FPG (mg/dL)/2], and the atherogenic index of plasma (AIP) was log10[triglycerides (mmol/L)/HDL-C (mmol/L)].

Covariates were selected a priori from established demographic and metabolic determinants of carotid atherosclerosis rather than data-driven stepwise selection. Model 0 was unadjusted; Model 1 adjusted for age and sex; Model 2 additionally for WHR and eGFR; and the prespecified primary Model 3 additionally adjusted for FPG, ln(triglycerides), HDL-C, and LDL-C (eight covariates in total). BMI was not entered concurrently with WHR and was examined in a replacement sensitivity analysis. Because fasting glucose and triglyceride and lipoprotein concentrations may lie partly on the metabolic pathway through which fatty-acid composition influences arterial structure, the Model 3 estimate is a conditional direct association after these pathway variables are held fixed rather than a total effect, and it is consequently conservative; the sequential Models 0–2 show the gradient toward this estimate (Supplementary Tables S2 and S3). Alternative models replaced the Model 3 metabolic block, one at a time, with TyG, AIP, ApoB or ApoA-I, or they replaced WHR with BMI as sensitivity analyses. Continuous blood pressure and detailed smoking history were not recorded in the study dataset and could not enter the adjustment set; their role as unmeasured potential confounders is examined in the Limitations section.

### 2.6. Statistical Analysis

#### 2.6.1. Primary Panel Regression and Multiplicity

The independent unit was one participant. Continuous variables are reported as mean ± standard deviation (SD) or median (interquartile range [IQR]) and categorical variables as *n* (%), with Kruskal–Wallis or chi-squared tests for unadjusted between-category comparisons; the max-CWT comparison in Table 1 is reported descriptively because max-CWT itself defined the severity categories, and unadjusted fatty-acid distributions were compared without multiplicity correction. For the primary outcome, ln(max-CWT), each fatty-acid measure was entered separately into an ordinary least squares model with heteroscedasticity-consistent type 3 (HC3) standard errors. Exposures were standardised to one sample SD, so *β* is the adjusted difference in ln(max-CWT) per 1-SD higher measure; aggregate totals, components and ratios were never entered simultaneously. The prespecified primary multiplicity standard was Benjamini–Hochberg false discovery rate correction pooled across all 16 tests [47], which needs no grouping of hypotheses. Because an exploratory grouping was defined during analysis and was not prospectively registered, we also report a more liberal within-family correction across three mutually exclusive families (four class totals, two ratios, ten individual/composite measures) solely to show how inference depends on the grouping; primary conclusions rest on the pooled standard. Variance inflation factors were inspected, and observations with Cook’s distance > 4/n were flagged but retained, with leave-one-participant-out (LOPO) refitting quantifying their influence. To quantify robustness to unmeasured confounding for the odds ratio outcomes, we computed E-values, that is, the minimum risk-ratio strength an unmeasured confounder would need with both the exposure and the outcome to explain away an estimate [48]. Because E-values are defined on the risk-ratio scale, whereas the vascular outcomes are common, odds-ratio-based E-values are exploratory approximations that may overstate robustness to unmeasured confounding; E-values for ordered severity pertain to that supporting outcome rather than to the continuous primary outcome. For each linear fit, we report the model R^2^ and adjusted R^2^, together with the increment in R^2^ over the covariate-only model at the same adjustment level, which quantifies the variance uniquely attributable to the fatty-acid measure.

#### 2.6.2. Composition-Aware Analysis

The *D* = 26 parts were expressed in the canonical orthonormal sequential pivot-ILR form [36,37]. For coordinate *j*, *z_j_* = √[(*D* − *j*)/(*D* – *j* + 1)] × {ln(*x_j_*) − mean[ln(*x*_*j*+1_, …, *x_D_*)]} on the closed composition [36,37]. All 25 standardised coordinates entered jointly with the Model 3 covariates, and the overall composition–outcome association used the HC3-robust Wald test of the 25 coefficients. Component-specific re-pivoted first-ILR contrasts compared each retained part with the geometric mean of the other 25 parts (one model at a time), with Benjamini–Hochberg q values across the 25 contrasts; centred log-ratio (CLR) models that omitted other_FA served as sensitivity. These CoDA analyses were exploratory and separate from the primary 16-measure families, and every component estimate is a relative-composition contrast rather than an effect of absolute concentration. The joint 25-coordinate model (residual degrees of freedom, 196) was additionally assessed for parameterisation burden and overfitting using variance inflation factors, repeated 10-fold cross-validation of out-of-sample R-squared for the covariate-only, single-exposure and full-composition models, and a 1000-permutation test of the global Wald statistic.

#### 2.6.3. Secondary, Supporting and Sensitivity Analyses

Secondary analyses used Model 3 median regression (*τ* = 0.50), restricted cubic splines for the five within-family FDR-flagged measures (knots at the 10th, 50th and 90th percentiles; nominal, post-selection *p* values), and fractional-logit regression for stenosis expressed as a 0–1 proportion (odds ratios describe the conditional-mean stenosis proportion, not the presence of stenosis). Cumulative-logit regression addressed ordered severity derived from max-CWT thresholds, treated as a supporting rather than an independent outcome; the proportional-odds assumption was checked with global and threshold-specific paired-score Wald tests. An exploratory contextual Benjamini–Hochberg calculation pooled, for each exposure, the *p* values across the three vascular outcomes (ln(max-CWT), stenosis proportion and ordered severity) without redefining the primary ln(max-CWT) findings. Targeted models retained the Model 3 covariates and additionally added EPA, DHA or both to ALA, AA to LA, and SCD1-16 or stearic acid to oleic-acid models; separate exploratory models fitted SCD1-16 and ln(SCD1-18), with Benjamini–Hochberg correction across these two derived-index tests. These targeted and derived-index models were conditional sensitivity analyses and were not used to estimate mediation, conversion efficiency, enzyme activity or metabolic flux. Unless stated otherwise, all tests were two-sided with nominal significance at *p* < 0.05, and analyses used Python 3.14.7, pandas 3.0.5, SciPy 1.18.1, statsmodels 0.15.0 and matplotlib 3.11.1.

## 3. Results

### 3.1. Participant Characteristics and Ultrasound Reproducibility

The 230 participants comprised 109 men (47.4%); 33, 132 and 65 were classified as mild, moderate and severe, respectively (Table 1). Median age was 55.0 years (IQR 48.0–62.0), median BMI 24.6 kg/m^2^ (IQR 22.40–26.68) and median WHR 0.90 (IQR 0.86–0.94). Median max-CWT rose from 1.34 mm (IQR 1.20–1.47) to 1.80 mm (IQR 1.66–2.09) and 3.01 mm (IQR 2.64–3.38) across categories, and median stenosis rose in parallel (13.05%, 22.36% and 34.40%; *p* < 0.001); age, eGFR and fasting glucose also differed across categories (*p* = 0.014, 0.006 and 0.001), whereas no other baseline variable did (Table 1). Because max-CWT defined the categories, its *p* value is descriptive. Intra-observer agreement was high for both max-CWT (n = 78; ICC(2,1) = 0.997, 95% confidence interval (CI) 0.991–0.999; relative SD 2.27%) and stenosis (n = 74; ICC(2,1) = 0.977, 95% CI 0.946–0.991; relative SD 8.02%). Mean differences were 0.054 mm (second readings slightly higher; paired *t* test *p* < 0.001) and 0.22 percentage points (*p* = 0.357; Supplementary Table S4).

### 3.2. DBS Fatty-Acid Measures Across Severity Categories

**Figure 2:**
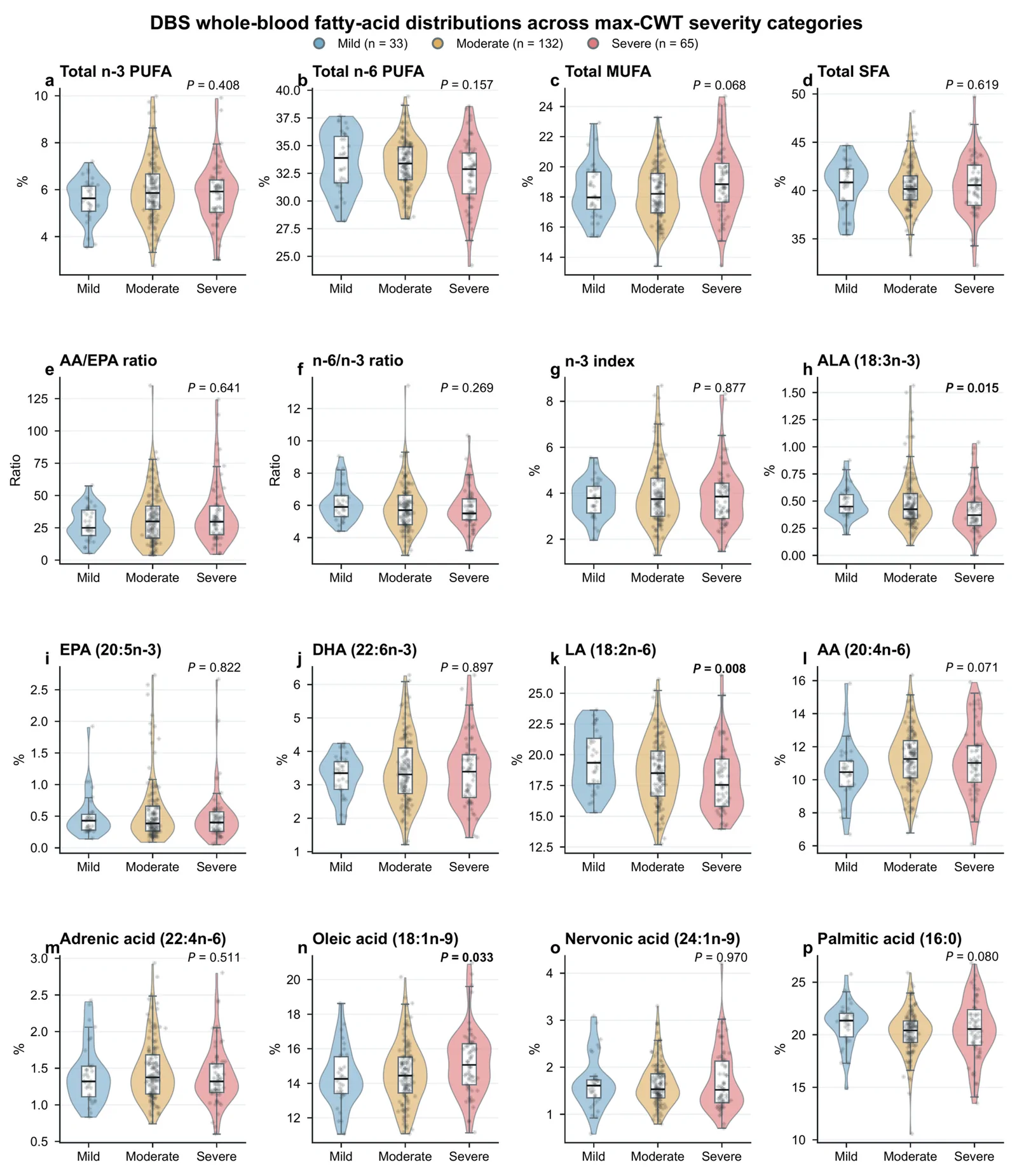
Distribution of the 16 DBS whole-blood fatty-acid measures across study-defined max-CWT severity categories (mild ≤ 1.5 mm, n = 33; moderate > 1.5 to <2.5 mm, n = 132; severe ≥ 2.5 mm, n = 65). Violin plots show unadjusted distributions with median and IQR marked; *p* values are from unadjusted Kruskal–Wallis tests. Except for the AA/EPA and *n*-6/*n*-3 ratios, measures are percentages of total identified fatty-acid peak area.

Three of the 16 measures differed across categories before adjustment (Figure 2; Supplementary Table S5). Median ALA fell from 0.45% (IQR 0.39–0.56) to 0.43% (IQR 0.34–0.57) and 0.37% (IQR 0.28–0.49) (*p* = 0.015), and mean LA fell from 19.60 ± 2.40% to 18.56 ± 2.64% and 17.96 ± 2.63% (*p* = 0.008); median oleic acid rose from 14.26% (IQR 13.41–15.54) to 14.45% (IQR 13.44–15.52) and 15.06% (IQR 13.92–16.29) (*p* = 0.033). EPA did not differ (*p* = 0.822), and no other measure showed a nominal difference.

### 3.3. Adjusted Associations with max-CWT and Model Robustness

**Figure 3:**
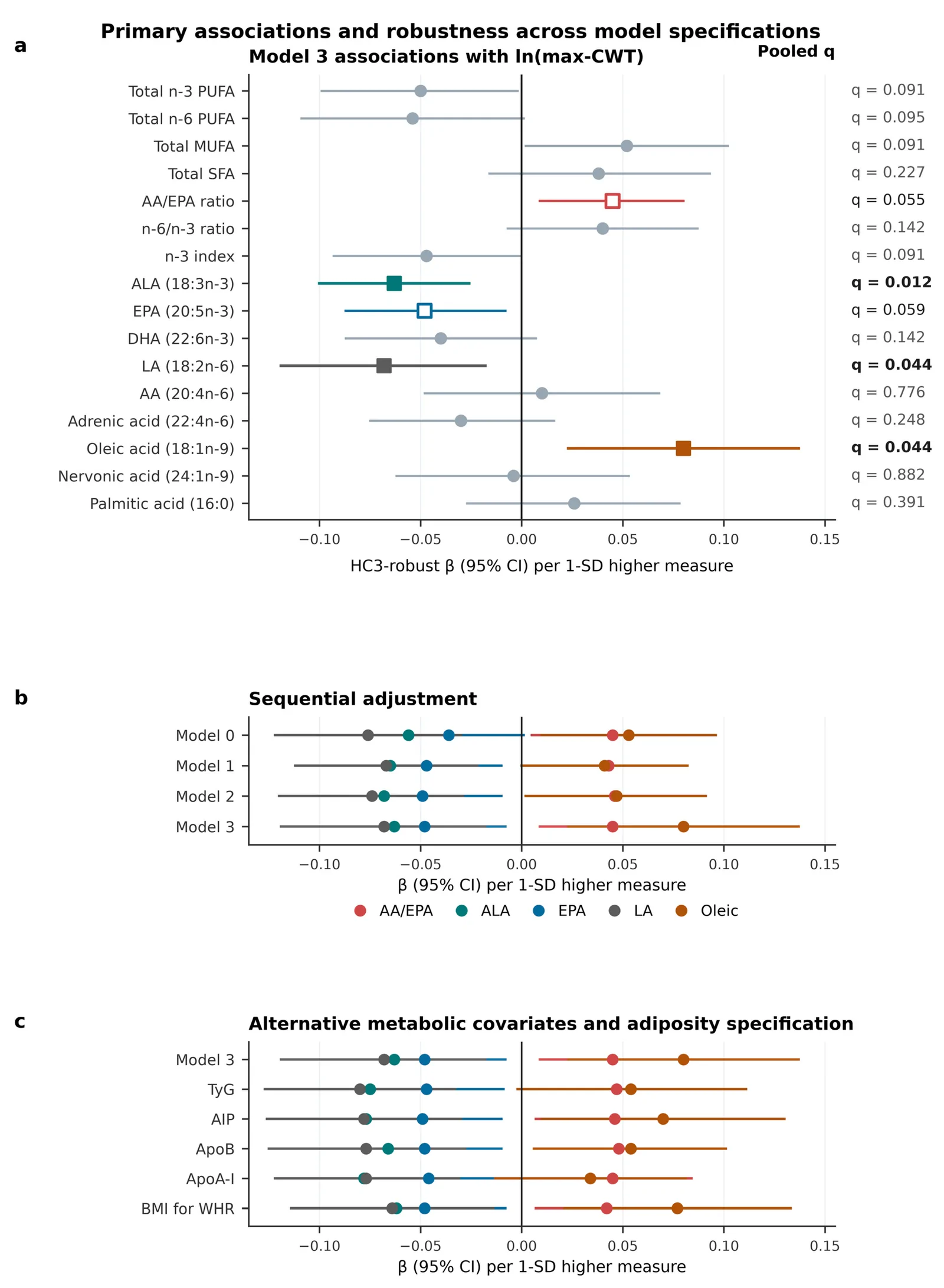
Primary associations with ln(max-CWT) and robustness across model specifications. (**a**) Model 3 per-1-SD associations for all 16 measures, with pooled 16-test q values listed at the right: filled squares denote measures significant under the prespecified pooled FDR standard (ALA, LA, oleic acid), open squares denote measures significant only under the exploratory within-family correction (EPA, AA/EPA ratio), and grey circles denote measures meeting neither threshold; (**b**) sequential Models 0–3; (**c**) alternative-covariate models for the five within-family FDR-flagged measures, including the BMI-for-WHR substitution. Points are HC3-robust *β* coefficients and bars are 95% CIs; all 230 participants contributed to every model.

Under the prespecified pooled Benjamini–Hochberg correction across all 16 tests (the primary multiplicity standard), 3 measures retained q < 0.05 in Model 3 (Figure 3a; Table 2). Per 1-SD higher exposure, ALA (*β* = −0.063, 95% CI −0.100–−0.026; *p* < 0.001; pooled q = 0.012) and LA (*β* = −0.068, 95% CI −0.119–−0.018; *p* = 0.008; pooled q = 0.044) were inversely and oleic acid (*β* = 0.080, 95% CI 0.023–0.137; *p* = 0.006; pooled q = 0.044) positively associated with ln(max-CWT). Under the more liberal exploratory within-family correction, two further measures reached its threshold but not the pooled standard: EPA (*β* = −0.048, 95% CI −0.087–−0.008; *p* = 0.018; within-family q = 0.046; pooled q = 0.059, inverse) and the AA/EPA ratio (*β* = 0.045, 95% CI 0.009–0.080; *p* = 0.014; within-family q = 0.027; pooled q = 0.055, positive); the other 11 measures met neither threshold.

All five within-family FDR-flagged estimates kept their direction across Models 0–3, and the AA/EPA ratio, ALA, EPA and LA remained nominally significant in every alternative metabolic-adjustment model (Figure 3b; Supplementary Tables S2 and S3). Oleic acid stayed positive but lost nominal significance after adjustment for the triglyceride–glucose index (*β* = 0.054, 95% CI −0.002–0.111; *p* = 0.058) or ApoA-I (*β* = 0.034, 95% CI −0.013–0.081; *p* = 0.158); replacing WHR with BMI preserved direction and nominal significance for all five. In LOPO refitting, direction was retained in all 230 refits for four measures and in 229/230 for EPA, and the largest relative change in |*β*| was 10.8–17.7%; the maximum variance inflation factor was 2.14 (Supplementary Tables S6 and S7). The eight Model 3 covariates alone accounted for 19.5% of the variance in ln(max-CWT) (adjusted R^2^ = 0.166); adding any single fatty-acid measure gave a full-model R^2^ of 0.195–0.222, and the increment in R^2^ over the covariate-only fit was largest for the three pooled-FDR signals—oleic acid (2.65%), ALA (2.43%) and LA (2.25%)—but was below 0.1% for the null AA and nervonic acid estimates (Supplementary Table S2).

### 3.4. Composition-Aware Analysis of the Full Profile

After pooling the 12 species detected in fewer than 95% of participants into other_FA, the 26-part composition yielded 25 pivot-ILR coordinates (Supplementary Table S1). The global Wald test of the full composition was significant (F(25,196) = 2.26; *p* = 0.0011; Table 3; Supplementary Table S8), indicating that overall relative DBS composition, not only selected species, was associated with ln(max-CWT). In component-specific re-pivoted contrasts, ALA was inversely (*β* = −0.079, 95% CI −0.118–−0.041; *p* = 0.000053; q = 0.0013) and oleic acid positively (*β* = 0.088, 95% CI 0.035–0.142; *p* = 0.0013; q = 0.0159) associated; these were the only two component contrasts to survive FDR. EPA was inverse at the nominal level only (*β* = −0.047, *p* = 0.0248; q = 0.2066), while LA retained its inverse direction but was attenuated and non-significant (*β* = −0.032, 95% CI −0.082–0.019; *p* = 0.2231; q = 0.4648). Marginal CLR estimates matched the re-pivoted balances to numerical precision (Supplementary Table S9).

**Figure 4:**
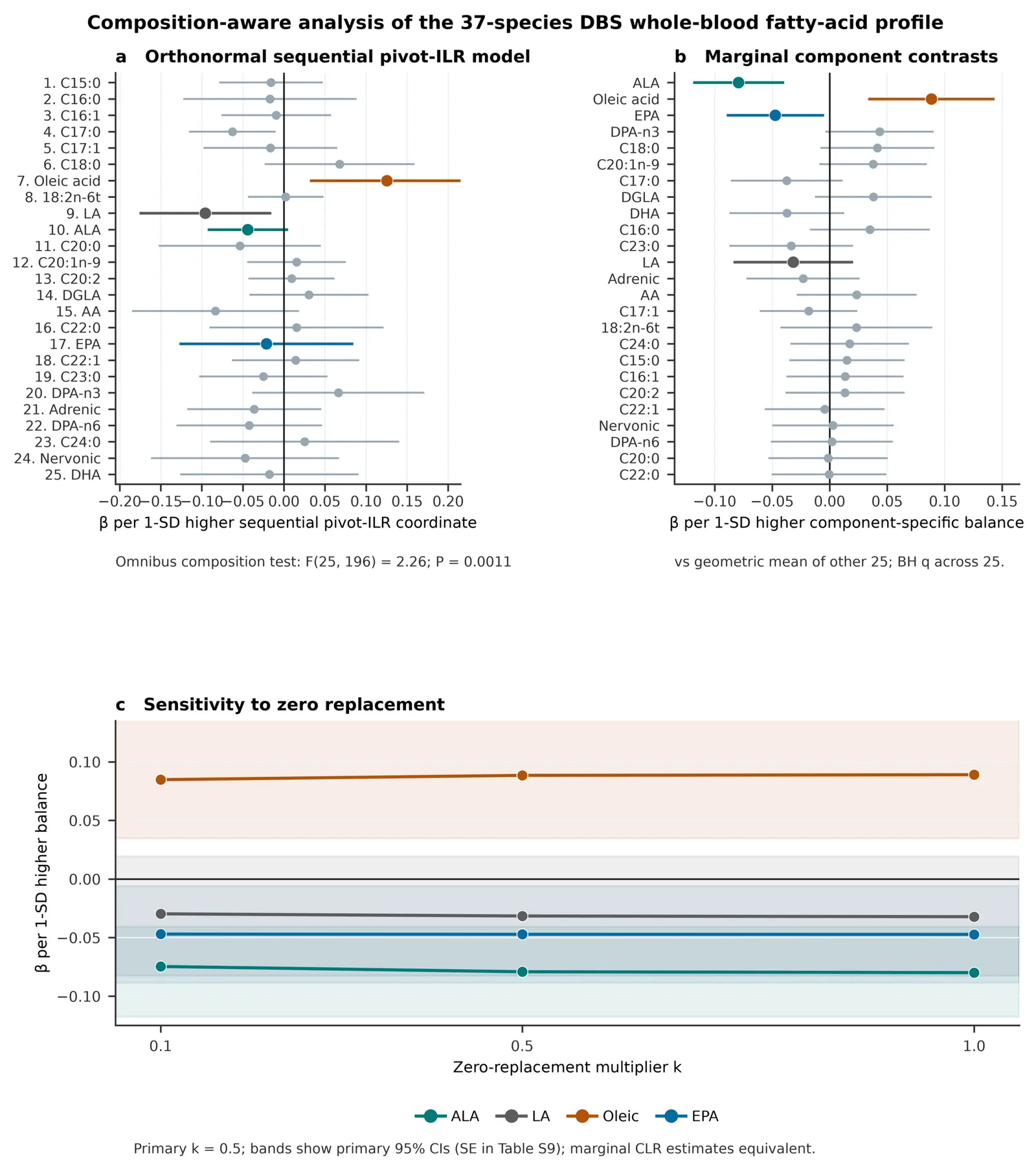
Composition-aware analysis of the 37-species DBS profile (26-part composition). (**a**) The 25 canonical sequential-pivot ILR coordinates; (**b**) component-specific re-pivoted first-ILR contrasts for the 25 retained parts; (**c**) ALA, oleic-acid and EPA contrasts at zero-replacement k = 0.1, 0.5, and 1.0. Points are per-1-SD HC3-robust Model 3 estimates and bars 95% CIs. The global 25-coordinate test was F(25,196) = 2.26, *p* = 0.0011.

The global test remained significant across zero-replacement constants k = 0.1, 0.5, and 1.0 (F = 2.59, 2.26 and 2.13; *p* = 0.00013, 0.0011 and 0.0022), with stable directions and magnitudes: ALA and oleic acid remained FDR-significant, EPA inverse at the nominal level, and LA inverse but non-significant at every constant; CLR models reproduced the omnibus and component estimates (Supplementary Tables S9 and S10; Figure 4). Two routes that parameterise the data very differently thus converged on inverse ALA and positive oleic acid contrasts, with LA being concordantly inverse. With 34 estimated parameters (residual degrees of freedom, 196; maximum variance inflation factor 5.57), the omnibus association was corroborated by a 1000-permutation test of the global Wald statistic (*p* = 0.003), but repeated 10-fold cross-validation showed no out-of-sample gain for the full 25-coordinate model over the 8 covariates alone (cross-validated R-squared 0.075 versus 0.123); adding prespecified single panel exposures, and the 3 pooled-FDR signals jointly, instead, raised cross-validated R-squared (to 0.144–0.146 and 0.166). The omnibus test therefore supports an overall compositional association, whereas component-level inference rests on the sparse prespecified panel, in line with the exploratory role assigned to the composition route (Supplementary Table S19). Because cross-validated prediction did not improve, the significant omnibus result should be read as evidence of an overall compositional association rather than of predictive or discrimination gain.

### 3.5. Robustness and Exposure–Response Shape

**Figure 5:**
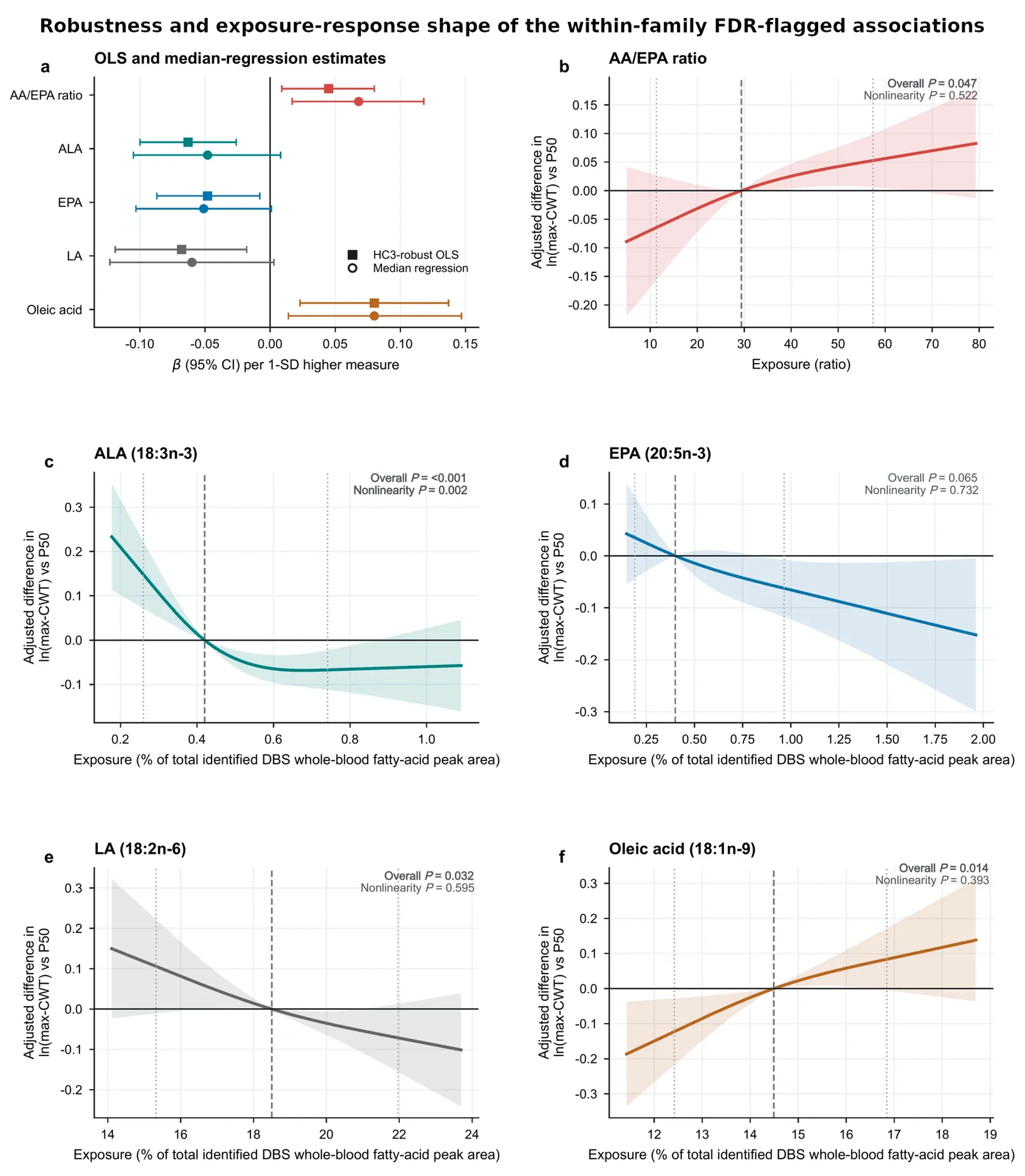
Robustness and observed exposure–response shape of the five within-family FDR-flagged associations. (**a**) HC3-robust OLS compared with robust-covariance median-regression estimates; (**b**–**f**) exploratory restricted cubic splines on the raw exposure scale (knots at the 10th, 50th and 90th percentiles), with adjusted predictions, 95% CIs, and the sample median as reference.

In Model 3 median regression, all five within-family FDR-flagged associations kept their direction (Figure 5a; Supplementary Table S11): the AA/EPA ratio (*β* = 0.068, 95% CI 0.017–0.118; *p* = 0.009) and oleic acid (*β* = 0.080, 95% CI 0.014–0.147; *p* = 0.018) remained significant, whereas the inverse ALA (*β* = −0.048, 95% CI −0.105–0.008; *p* = 0.094), EPA (*β* = −0.051, 95% CI −0.103–0.001; *p* = 0.055) and LA (*β* = −0.060, 95% CI −0.123–0.003; *p* = 0.060) estimates had intervals including the null. Restricted cubic splines showed nominal overall associations for the AA/EPA ratio (*p* = 0.047), ALA (*p* < 0.001), LA (*p* = 0.032), and oleic acid (*p* = 0.014) but not EPA (*p* = 0.065) (Figure 5b–f; Supplementary Table S12). ALA showed nonlinearity (*p* = 0.002), with adjusted ln(max-CWT) falling more steeply across its lower-to-middle range and then levelling off; the other four were approximately linear (nonlinearity *p* = 0.393–0.732).

### 3.6. Carotid Stenosis and Ordered Severity

**Figure 6:**
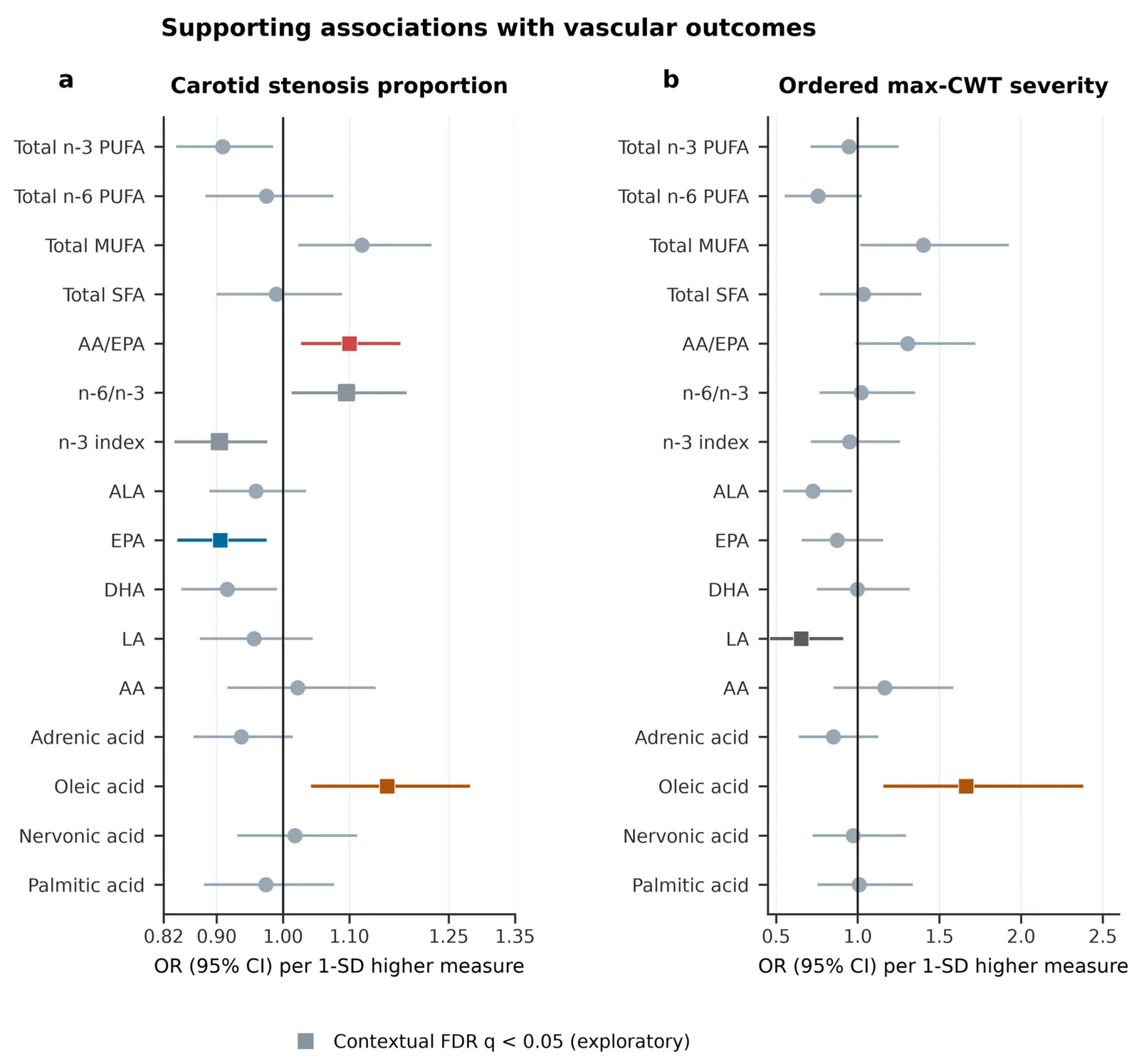
Supporting vascular outcomes. (**a**) Fractional-logit ORs for the conditional-mean stenosis proportion; (**b**) cumulative-logit ORs for classification into a higher study-defined max-CWT severity category. Points are per-1-SD Model 3 ORs and bars are 95% CIs (filled symbols, contextual FDR q < 0.05); all 230 participants contributed.

For the conditional-mean stenosis proportion (fractional logit; this models the proportion, not the odds of having stenosis), higher AA/EPA (OR 1.100, 95% CI 1.030–1.175; q = 0.028) and *n*-6/*n*-3 (OR 1.096, 95% CI 1.015–1.184; q = 0.038) ratios and higher oleic acid (OR 1.157, 95% CI 1.045–1.280; q = 0.035) were associated with greater stenosis, whereas the *n*-3 index (OR 0.904, 95% CI 0.838–0.974; *p* = 0.008; q = 0.035) and EPA (OR 0.905, 95% CI 0.843–0.973; *p* = 0.007; q = 0.035) were inverse (Figure 6a; Supplementary Table S13). In ordered-severity models, LA was associated with lower odds of a more severe category (OR 0.653, 95% CI 0.473–0.901; q = 0.036) and oleic acid with higher odds (OR 1.664, 95% CI 1.168–2.370; q = 0.035; Figure 6b). Proportional-odds diagnostics showed no departure from the assumption (global *p* = 0.151–0.577; exposure-specific *p* = 0.651 for LA and 0.096 for oleic acid; Supplementary Table S14). E-values for the point estimates were 2.72 (with a lower confidence bound of 1.61) for oleic acid’s ordered severity and 2.43 (1.46) for LA, and ranged from 1.42 to 1.58 (with lower bounds of 1.14–1.26) across the stenosis proportion odds ratios, quantifying the joint exposure–outcome association an unmeasured confounder would need to nullify each estimate [48].

### 3.7. Targeted Related-Measure and Derived-Index Analyses

**Figure 7:**
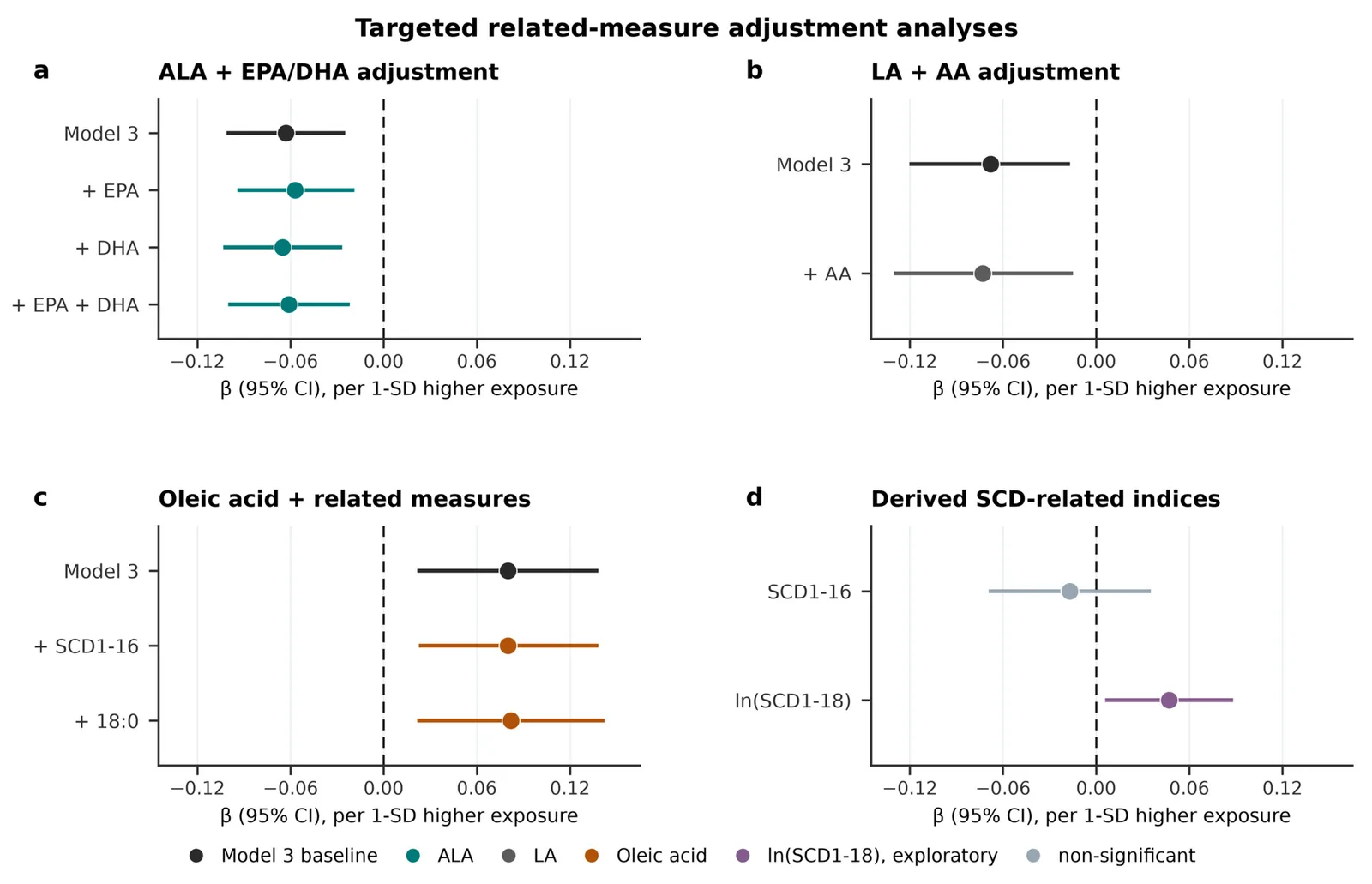
Targeted related-measure adjustment and derived indices. (**a**) ALA additionally adjusted for EPA, DHA or both; (**b**) LA adjusted for AA; (**c**) oleic acid adjusted for SCD1-16 or stearic acid (18:0); (**d**) separate models for SCD1-16 and ln(SCD1-18). Points are per-1-SD HC3-robust β coefficients, and bars are 95% CIs; all models retained the Model 3 covariates and all 230 participants. SCD1-18 is a compositional proxy that contains oleic acid in its numerator (Spearman ρ = 0.831) and does not measure SCD1 enzyme activity or metabolic flux.

The inverse ALA estimate changed little after additional adjustment for EPA (*β* = −0.057, 95% CI −0.093–−0.020; *p* = 0.002), DHA (*β* = −0.065, 95% CI −0.102–−0.028; *p* < 0.001) or both (*β* = −0.061, 95% CI −0.099–−0.023; *p* = 0.002), and the inverse LA estimate was likewise stable after adjustment for AA (*β* = −0.073, 95% CI −0.129–−0.016; *p* = 0.012). The positive oleic-acid estimate was essentially unchanged after adjustment for SCD1-16 (*β* = 0.080, 95% CI 0.024–0.137; *p* = 0.005) or stearic acid (*β* = 0.082, 95% CI 0.023–0.141; *p* = 0.006). ln(SCD1-18) was positively associated (*β* = 0.047, 95% CI 0.007–0.087; *p* = 0.021, q = 0.043) but SCD1-16 was not (*β* = −0.017, 95% CI −0.068–0.034; *p* = 0.510); because SCD1-18 contains oleic acid in its numerator and correlates strongly with it (Spearman *ρ* = 0.831), it is not treated as independent corroboration (Figure 7; Supplementary Tables S15–S18). Targeted estimates for ALA, LA, and oleic acid retained direction and nominal significance in all 230 LOPO refits, and ln(SCD1-18) did so in 228/230.

## 4. Discussion

### 4.1. Principal Findings

We are not aware of a previous study that related a DBS whole-blood fatty-acid profile to a structural vascular endpoint or that cross-checked that question within one cohort through two differently parameterised analytic routes. This study positions low-volume DBS whole-blood profiling as an objective nutritional–metabolic phenotype in a Chinese metabolic-risk cohort and tests that phenotype against ultrasound-defined carotid wall burden. Two analytic routes with different parameterisations—a prespecified 16-measure panel under a common covariate framework with explicit FDR control, and a closed 26-part composition analysed by log-ratio methods—converged on the same signal set. Inverse ALA and positive oleic-acid contrasts were recovered by both routes and were the most consistent; LA was inverse across the conventional, severity-gradient, ordered and spline analyses but attenuated in the closed composition; EPA and the AA/EPA ratio were weaker, family-level signals. The directions agree with prospective and surgical cohorts elsewhere, which provides external corroboration against a chance finding. Per 1-SD higher measure, ALA and LA corresponded to a geometric-mean max-CWT about 6–7% lower and oleic acid about 8% higher, or roughly 0.11–0.15 mm at the cohort median of 1.88 mm—two to three times the 0.054 mm systematic difference between repeat readings. These contrasts sit against a markedly *n*-6-dominant profile (total *n*-6 PUFA 33.09 ± 2.54%; median total *n*-3 PUFA 5.88%; median *n*-6/*n*-3 = 5.70 and AA/EPA = 29.40), which is characteristic of this East-Asian setting. Because every exposure is a relative percentage or ratio, the estimates describe within-profile compositional contrasts rather than absolute-concentration effects; for example, a higher relative oleic acid proportion can arise from more oleic acid itself or from reductions in other components, so this is neither an absolute-concentration nor a dose effect. Three pooled-FDR-robust contrasts (lower ALA and LA and higher oleic acid) and two weaker family-level signals (EPA and the AA/EPA ratio) jointly delineate one characteristic pattern.

The composition-aware analysis supplies the internal cross-check. Because the 37 fatty-acid percentages share one fixed total, a rise in any component necessarily lowers the relative contribution of the others—a constraint that measure-by-measure regression cannot represent. Without changing the covariate model, the global ILR test was significant (F(25,196) = 2.26; *p* = 0.0011), and re-pivoted contrasts localised the profile-level association to ALA (*β* = −0.079; q = 0.0013) and oleic acid (*β* = 0.088; q = 0.0159) in the same directions as the conventional panel. LA is a large, strongly covarying part, and its one-versus-rest log-ratio removes variation shared with the remaining *n*-6 elongation products; it therefore tests a narrower, more conservative relative substitution contrast than its conventional percentage, which accounts for the attenuation (*β* = −0.032; *p* = 0.223) without any reversal of direction. The inference was stable to the zero-replacement constant and reproduced by CLR models to numerical precision, which supports the stability of the pattern to how the closed data are parameterised.

### 4.2. Interpretation of the Individual Fatty-Acid Signals

ALA carried the strongest inverse signal under both FDR schemes and illustrates the agreement between internal and external evidence. Its spline declined more steeply across the lower-to-middle observed range and then levelled off (nonlinearity *p* = 0.002), suggesting the testable hypothesis that, among adults above the structural involvement threshold with ALA below the cohort median of 0.42%, higher ALA status tracks with lower wall burden. Adjustment for EPA, DHA or both changed the estimate little, so the long-chain *n*-3 percentages do not account for it. As the principal plant-derived *n*-3 PUFA, whose conversion to EPA and DHA is limited [21], ALA enters membrane phospholipids and influences vascular biology through oxylipin and inflammatory pathways [22]. Higher dietary ALA has been prospectively associated with lower cardiovascular mortality [49], *n*-3 status or intake with lower carotid plaque burden, progression and intima–media thickness [29,30,50]; ALA interventions in adults with overweight or obesity have been associated with modest cardiometabolic improvement [23], including larger long-chain *n*-3 gains in those with low baseline EPA [24]. Reproducing this direction in an internal DBS compositional contrast extends the evidence to a Chinese metabolic-risk cohort [27,28].

LA was the other inverse signal to pass pooled FDR and shows parallel concordance. Higher LA was associated with lower ln(max-CWT), with an approximately linear gradient and lower odds of a more severe category (OR 0.653, 95% CI 0.473–0.901); adjustment for AA changed the estimate little, and median regression kept the inverse direction. As an essential *n*-6 PUFA embedded in membrane phospholipids, lipoprotein metabolism and lipid signalling [26,31,32], LA in this population derives mainly from seed oils, nuts and seeds, and replacing saturated-fat sources with LA-rich unsaturated foods is the relevant nutritional solution [31,33,34]. Externally, higher erythrocyte and plaque-phospholipid LA marked a lower prevalence of symptomatic carotid disease in an endarterectomy cohort [35]; an individual-level pooled analysis of 30 prospective cohorts linked higher LA biomarkers to lower incident cardiovascular disease, cardiovascular mortality, and ischaemic stroke [51]; and higher erythrocyte-membrane LA was associated with fewer carotid plaques in adults with type 1 diabetes [52]. The LA signal is graded below ALA and oleic acid in certainty but is externally corroborated and merits longitudinal replication.

Oleic acid was the clearest positive signal, concordant across continuous max-CWT (*β* = 0.080), stenosis (OR 1.157), and ordered severity (OR 1.664). Its source requires care. Chinese edible-oil patterns differ from olive oil-rich Mediterranean diets, so a whole-blood oleic acid percentage is not a marker of olive oil intake [27]; high-oleic oils can raise dietary oleic acid [33,34,53,54], but oleic acid is also generated endogenously from stearic acid through SCD1-related desaturation under hepatic lipogenesis and insulin-resistant states [55,56,57]. Two findings favour a metabolic contribution: adjustment for SCD1-16 or stearic acid left the estimate unchanged, while SCD1-16 itself showed no association, and replacing the metabolic block with the triglyceride–glucose index or ApoA-I attenuated but did not reverse it. Mechanistically, oleic-acid exposure promoted adipogenesis through altered AKT2/LXRα signalling [58], plasma MUFA dominated by oleic acid has been related to human obesity [53], and reduced oleic-acid availability under SCD1 deficiency shifted thymocyte differentiation toward Treg and away from Th17 programmes [59]. Oleic acid is thus the positive counterpart to the inverse plant-derived PUFAs, although its dietary and metabolic origins remain to be separated. A whole-blood oleic acid percentage is a circulating compositional measure that combines dietary oleic intake with endogenous SCD1-mediated synthesis; it is not a biomarker of olive oil intake, and the positive association does not imply that higher olive oil or dietary oleic consumption would increase carotid wall burden. Because diet, cooking oil type, and supplement use were not comprehensively assessed, no intake-based inference can be drawn from this compositional signal.

EPA and the AA/EPA ratio add complementary, lower-certainty signals. EPA was inversely associated with max-CWT and stenosis, and the AA/EPA ratio positively with max-CWT. EPA enters membrane phospholipids and yields lipid mediators that regulate inflammation and its resolution [60,61,62], while the AA/EPA ratio indexes the balance of two bioactive pools and is a research surrogate for inflammatory tendency [2,63]. Because AA alone was not associated, the positive ratio contrast may reflect variation in the EPA denominator rather than an independent AA effect. Both measures met family-specific but not pooled FDR and are best retained as complementary candidates ranked below ALA, LA, and oleic acid.

### 4.3. Nutritional and Clinical Interpretation

These findings translate into clinical nutrition at two complementary levels. Individual measures track structural carotid change: lower ALA and LA with higher oleic acid and AA/EPA accompanied greater wall burden across continuous, ordered, and stenosis outcomes under one covariate backbone, with directional stability in LOPO refitting. At the profile level, the 16-measure DBS panel characterises overall nutritional–metabolic state rather than a single nutrient. Unlike a questionnaire, which records reported intake, and routine lipid biochemistry, which does not resolve fatty-acid composition, DBS measures internal composition after absorption and endogenous metabolism and adds a dimension absent from both [1,2,3,4,5,6]. Its small capillary volume allows repeated collection, and published work documents DBS repeatability [8,38], interlaboratory reproducibility [43], sensitivity to dietary change [7,8], short-term temporal responsiveness [9] and a broader validation base for cardiometabolic assays [10]. Paired baseline and follow-up profiles can therefore document intervention-related compositional shifts alongside a structural ultrasound outcome [4,5,6,8,9,10,11]. The three pooled-FDR-robust contrasts (lower ALA and LA and higher oleic acid), together with the weaker EPA and AA/EPA signals, describe a characteristic pattern against an *n*-6-dominant background. These cross-sectional, single-measure data establish an association, not that changing dietary intake would alter max-CWT; the pattern is a candidate nutritional–metabolic phenotype that warrants prospective investigation, and longitudinal cohorts with repeated DBS sampling and randomised dietary interventions are required before any nutritional recommendation can be derived. Its incremental value beyond conventional indicators should likewise be quantified prospectively.

### 4.4. Strengths and Limitations

The principal strength is the pairing, within one well-characterised cohort, of a prespecified panel with explicit family and pooled FDR control and a formal composition-aware analysis, reinforced by high intra-observer ultrasound reproducibility (ICC(2,1) = 0.997) and concordance with external prospective cohorts. Several boundaries define the next steps, and two selection issues frame the scope of inference. First, because eligibility required max-CWT of at least 1.0 mm, a threshold on the outcome phenotype itself, the associations are conditional on established structural involvement; such conditioning can induce collider (selection) bias whose direction cannot be determined from these data, and the results must not be extrapolated to adults free of structural involvement or to the general population. Second, the cross-sectional, single-centre design and one-time DBS measurement do not establish temporal order; whether serial DBS sampling can support longitudinal, repeated-measure assessment is not established by this single-measurement study. The estimates therefore describe within-population gradients in wall burden among adults with structural involvement, not contrasts against adults free of structural involvement, a separate question requiring a population-based sample; the composite max-CWT endpoint also combines IMT thickening and focal plaque without per-segment separation, as described in the Methods section. One certified sonographer acquired and read every scan, so inter-observer reproducibility could not be assessed despite the high intra-observer agreement; multi-reader, dual-reading reproducibility is needed in future work. Several factors that can influence both fatty-acid composition and carotid wall thickness were not comprehensively measured: continuous blood pressure and detailed smoking history; statin and other lipid- or blood-pressure-lowering therapy; alcohol intake; physical activity; dietary intake and cooking-oil type; and omega-3 or fish oil supplementation. Each can act on both sides of the association: fish oil supplements directly raise circulating EPA and DHA, while supplement users often share other health behaviours. Statins alter lipoprotein metabolism, and smoking, blood pressure, and alcohol affect both hepatic lipid handling and arterial wall progression, so residual confounding in either direction cannot be excluded. Pairing DBS with a validated questionnaire and a medication inventory in future studies would separate internal composition from reported intake. Finally, because dietary patterns and edible oil use differ markedly across populations, these findings may not generalise directly to Western populations or to individuals without carotid structural involvement. E-value analysis quantifies the required magnitude: for the ordered-severity odds ratios, the point-estimate E-values were 2.72 for oleic acid and 2.43 for LA (with lower confidence bounds of 1.61 and 1.46, respectively) and 1.42–1.58 across the stenosis outcomes, so an unmeasured confounder would need risk-ratio associations of at least this size with both the fatty-acid profile and the outcome—magnitudes comparable to typical adjusted associations of blood pressure or smoking with carotid structure—to substantially attenuate the estimates, subject to the odds-ratio and ordered-outcome caveats noted in the Methods section [48]. Without a paired plasma or erythrocyte sample, DBS values cannot be exchanged for plasma or membrane measures, and a protocol-specific cross-matrix and stability sub-study would complement the published context [10,41,42,43]. The exploratory within-family correction is less conservative than the prespecified pooled correction that defines the primary findings and is reported only to show grouping sensitivity. None of these boundaries reverses the internally convergent, externally concordant directions; together, they mark a clear route from a candidate fatty-acid pattern to prospective and intervention testing.

## 5. Conclusions

In Chinese adults with carotid structural involvement and metabolic-risk features, low-volume DBS whole-blood fatty-acid profiling provides an objective nutritional–metabolic readout that integrates dietary absorption with endogenous metabolism and complements both dietary questionnaires and routine lipid biochemistry. A prespecified 16-measure panel and a closed 26-part ILR/CLR analysis, used as two complementary routes, converged on the same contrasts: the overall composition was associated with max-CWT (F(25,196) = 2.26, *p* = 0.0011); inverse ALA and positive oleic-acid contrasts were recovered by both strategies; LA carried a concordant inverse direction attenuated only in the closed composition; and EPA and AA/EPA supplied weaker complementary signals, all in agreement with external prospective cohorts. Against the pronounced *n*-6 predominance of this cohort (median *n*-6/*n*-3 = 5.70 and AA/EPA = 29.40), three pooled-FDR-robust contrasts—lower ALA and LA and higher oleic acid—together with weaker complementary EPA and AA/EPA signals define a candidate nutritional–metabolic pattern associated with carotid wall burden in this structurally involved population. Within adults who already show carotid structural involvement, individual measures signal the degree of structural carotid change, while the profile may be a candidate for serial nutritional assessment in future longitudinal research, although, on the evidence of this single-measurement study, its within-cohort repeatability and responsiveness to dietary intervention remain to be established. By pairing DBS profiling with formal compositional analysis, this work offers a practical nutritional–metabolic phenotyping approach, a transferable dual-route cross-check design, and candidate signals for prospective evaluation; longitudinal cohorts and randomised dietary intervention trials with repeated DBS sampling are required before any nutritional recommendation can be made.

## Figures and Tables

**Table 1:** Participant characteristics across study-defined max-CWT severity categories.

Variable	Overall (n = 230)	Mild Group (n = 33)	Moderate Group (n = 132)	Severe Group (n = 65)	*p* Value
Demographic characteristics					
Age, years	55.00 (48.00–62.00)	52.00 (40.00–58.00)	55.00 (48.00–61.00)	56.00 (52.00–63.00)	0.014
Male sex, n (%)	109 (47.4)	14 (42.4)	58 (43.9)	37 (56.9)	0.190
Primary outcome					
max-CWT, mm	1.88 (1.61–2.58)	1.34 (1.20–1.47)	1.80 (1.66–2.09)	3.01 (2.64–3.38)	<0.001
Secondary outcome					
Luminal stenosis, %	23.76 (18.02–30.41)	13.05 (0.00–15.48)	22.36 (18.89–25.98)	34.40 (29.60–42.37)	<0.001
Anthropometric indices					
BMI, kg/m^2^	24.60 (22.40–26.68)	24.60 (23.00–25.70)	24.70 (22.27–26.62)	24.50 (22.90–26.80)	0.996
WHR	0.90 (0.86–0.94)	0.92 (0.86–0.95)	0.90 (0.87–0.94)	0.90 (0.86–0.93)	0.907
Biochemical indices					
ALT, U/L	22.00 (16.25–29.00)	21.00 (17.00–31.00)	22.00 (16.00–29.00)	23.00 (18.00–31.00)	0.851
Uric acid, μmol/L	272.00 (226.75–329.25)	252.00 (229.00–344.00)	275.50 (225.50–325.50)	268.00 (235.00–323.00)	0.850
eGFR, mL/min/1.73 m^2^	103.10 (97.45–109.40)	108.38 (100.59–117.02)	103.08 (97.60–108.74)	101.01 (95.88–106.19)	0.006
Triglycerides, mmol/L	1.24 (0.91–1.67)	1.27 (0.95–1.67)	1.19 (0.92–1.63)	1.24 (0.90–1.73)	0.950
TyG index	8.37 ± 0.58	8.33 ± 0.58	8.33 ± 0.58	8.48 ± 0.59	0.247
HDL-C, mmol/L	1.44 (1.25–1.68)	1.59 (1.31–1.71)	1.44 (1.25–1.75)	1.39 (1.20–1.58)	0.064
LDL-C, mmol/L	2.94 ± 0.84	3.05 ± 0.78	2.93 ± 0.80	2.90 ± 0.96	0.719
ApoA-I, g/L	1.58 (1.38–1.82)	1.68 (1.52–1.98)	1.56 (1.38–1.87)	1.60 (1.38–1.76)	0.263
ApoB, g/L	0.78 ± 0.22	0.80 ± 0.21	0.78 ± 0.21	0.77 ± 0.25	0.858
FPG, mmol/L	4.37 (3.80–5.01)	4.20 (3.80–4.55)	4.28 (3.64–4.82)	4.80 (4.11–5.47)	0.001
AIP	−0.05 (−0.25–0.08)	−0.09 (−0.31–0.06)	−0.05 (−0.25–0.08)	−0.04 (−0.22–0.11)	0.638

Data are mean ± SD, median (IQR) or n (%). Between-category comparisons used Kruskal–Wallis or chi-squared tests; the max-CWT *p* value is descriptive because max-CWT defined the categories, and no multiplicity correction was applied. Abbreviations: AIP, atherogenic index of plasma; ALT, alanine aminotransferase; ApoA-I, apolipoprotein A-I; ApoB, apolipoprotein B; BMI, body mass index; eGFR, estimated glomerular filtration rate; FPG, fasting plasma glucose; HDL-C, high-density lipoprotein cholesterol; IQR, interquartile range; LDL-C, low-density lipoprotein cholesterol; max-CWT, maximum carotid wall thickness; TyG, triglyceride–glucose index; WHR, waist-to-hip ratio.

**Table 2:** Model 3 associations of 16 DBS whole-blood fatty-acid measures with ln(max-CWT).

Fatty-Acid Group	Measure	β (95% CI)	*p* Value	Family-Specific Q Value	Pooled 16-Test q Value
Total fatty-acid classes	Total *n*-3 PUFA	−0.050 (−0.099–−0.002)	0.042	0.071	0.091
Total fatty-acid classes	Total *n*-6 PUFA	−0.054 (−0.109–0.001)	0.053	0.071	0.095
Total fatty-acid classes	Total MUFA	0.052 (0.002–0.102)	0.042	0.071	0.091
Total fatty-acid classes	Total SFA	0.038 (−0.016–0.093)	0.170	0.170	0.227
Fatty-acid ratios	AA/EPA ratio	0.045 (0.009–0.080)	0.014	0.027	0.055
Fatty-acid ratios	*n*-6/*n*-3 ratio	0.040 (−0.007–0.087)	0.096	0.096	0.142
Individual/composite measures	*n*-3 index	−0.047 (−0.093–−0.001)	0.046	0.091	0.091
Individual/composite measures	ALA (18:3n-3)	−0.063 (−0.100–−0.026)	<0.001	0.008	0.012
Individual/composite measures	EPA (20:5n-3)	−0.048 (−0.087–−0.008)	0.018	0.046	0.059
Individual/composite measures	DHA (22:6n-3)	−0.040 (−0.087–0.007)	0.098	0.163	0.142
Individual/composite measures	LA (18:2n-6)	−0.068 (−0.119–−0.018)	0.008	0.027	0.044
Individual/composite measures	AA (20:4n-6)	0.010 (−0.048–0.068)	0.728	0.809	0.776
Individual/composite measures	Adrenic acid (22:4n-6)	−0.030 (−0.075–0.016)	0.202	0.288	0.248
Individual/composite measures	Oleic acid (18:1n-9)	0.080 (0.023–0.137)	0.006	0.027	0.044
Individual/composite measures	Nervonic acid (24:1n-9)	−0.004 (−0.062–0.053)	0.882	0.882	0.882
Individual/composite measures	Palmitic acid (16:0)	0.026 (−0.027–0.078)	0.342	0.428	0.391

HC3-robust β coefficients and 95% CIs per 1-SD higher measure. The pooled q value across all 16 tests is the prespecified primary multiplicity standard; the family-specific q values (four aggregate-total, two ratio, ten individual/composite families) are an exploratory and more liberal grouping-level analysis. The study-defined *n*-3 index (EPA + DHA) is not the validated erythrocyte omega-3 index. Abbreviations: AA, arachidonic acid; ALA, alpha-linolenic acid; CI, confidence interval; DBS, dried blood spot; DHA, docosahexaenoic acid; EPA, eicosapentaenoic acid; FDR, false discovery rate; LA, linoleic acid; max-CWT, maximum carotid wall thickness; MUFA, monounsaturated fatty acid; PUFA, polyunsaturated fatty acid; SFA, saturated fatty acid.

**Table 3:** Composition-aware associations of the 37-species DBS whole-blood fatty-acid profile with ln(max-CWT).

Analysis	Coordinate or Test	β or F (95% CI)	*p* Value	BH q Value
Global composition	25-coordinate joint Wald test	F(25, 196) = 2.26	0.0011	—
Component-specific re-pivoted ILR	ALA (18:3*n*-3)	−0.079 (−0.118–−0.041)	0.000053	0.0013
Component-specific re-pivoted ILR	Oleic acid (18:1n-9)	0.088 (0.035–0.142)	0.0013	0.0159
Component-specific re-pivoted ILR	EPA (20:5*n*-3)	−0.047 (−0.088–−0.006)	0.0248	0.2066
Component-specific re-pivoted ILR	LA (18:2*n*-6)	−0.032 (−0.082–0.019)	0.2231	0.4648

HC3-robust Model 3 estimates per 1-SD higher standardised coordinate. The global test jointly evaluates the 25 sequential-pivot ILR coordinates; component-specific models re-pivoted one retained part against the geometric mean of the other 25 parts, with Benjamini–Hochberg q across the 25 contrasts. Estimates are relative-composition (not absolute-concentration) contrasts.

## Data Availability

The aggregate datasets supporting the conclusions of this article are included within the article and its Supplementary Files (source data underlying Figure 1, Figure 2, Figure 3, Figure 4, Figure 5, Figure 6 and Figure 7, the analysis tables (including compositional data outputs), and the ultrasound intra-observer reproducibility assessment). Individual-level participant data are not publicly available due to privacy and ethics restrictions; requests for de-identified data will be reviewed by the corresponding author and the relevant institutional review bodies, subject to applicable approvals. The statistical analysis code is available from the corresponding author upon reasonable request.

## Supplementary Materials

The following supporting information can be downloaded at: https://www.mdpi.com/article/10.3390/nu18213495/s1, Additional File S1 (Supplementary Tables S1–S20, workbook): Table S1, Construction and diagnostics of the 26-part DBS whole-blood fatty-acid composition; Table S2, Sequential associations with ln(max-CWT) across Models 0–3; Table S3, Alternative covariate and body size sensitivity analyses; Table S4, Intra-observer reproducibility of carotid ultrasound measurements; Table S5, Distribution of DBS whole-blood fatty-acid measures by max-CWT severity category; Table S6, Leave-one-participant-out analysis for the five within-family FDR-flagged measures; Table S7, Model 3 heteroscedasticity, collinearity and influence diagnostics; Table S8, Canonical sequential pivot-ILR analysis of the 26-part DBS whole-blood fatty-acid composition; Table S9, Component-specific one-versus-rest localization and CLR sensitivity analysis; Table S10, Sensitivity of composition-aware associations to the zero-replacement constant; Table S11, Model 3 median regression sensitivity analysis; Table S12, Restricted cubic spline analyses for the five within-family FDR-flagged measures; Table S13, Model 3 associations with carotid stenosis and ordered max-CWT severity; Table S14, Proportional-odds diagnostics for ordered max-CWT severity; Table S15, Spearman correlations among the 16 DBS whole-blood fatty-acid measures; Table S16, Targeted related-measure and derived SCD-index sensitivity analyses; Table S17, Correlations among pathway-related fatty acids and SCD indices; and Table S18, Leave-one-participant-out checks for targeted related-measure models; Table S19, Overfitting, cross-validation and permutation diagnostics for the 26-part compositional model; and Table S20, Exploratory stratification at an operational 1.5 mm threshold and three-outcome concordance. Additional File S2: STROBE reporting checklist.

## Abbreviations

Abbreviations used in this manuscript are listed below.

**Abbreviation**	**Definition**
AA	arachidonic acid
AA/EPA	arachidonic acid-to-eicosapentaenoic acid ratio
AIP	atherogenic index of plasma
AKT2	AKT serine/threonine kinase 2
ALA	alpha-linolenic acid
ALT	alanine aminotransferase
ApoA-I	apolipoprotein A-I
ApoB	apolipoprotein B
BH	Benjamini–Hochberg
BMI	body mass index
CI	confidence interval
CKD-EPI	Chronic Kidney Disease Epidemiology Collaboration
CLR	centered log-ratio
CoDA	compositional data analysis
DBS	dried blood spot
DHA	docosahexaenoic acid
DICOM	Digital Imaging and Communications in Medicine
ECST	European Carotid Surgery Trial
eGFR	estimated glomerular filtration rate
EPA	eicosapentaenoic acid
FAME	fatty-acid methyl ester
FDR	false discovery rate
FPG	fasting plasma glucose
GC-FID	gas chromatography with flame-ionisation detection
HC3	heteroscedasticity-consistent covariance estimator, type 3
HDL-C	high-density lipoprotein cholesterol
ICC	intraclass correlation coefficient
ILR	isometric log-ratio
IMT	intima–media thickness
IQR	interquartile range
ISO	International Organization for Standardization
LA	linoleic acid
LC–MS/MS	liquid chromatography–tandem mass spectrometry
LDL-C	low-density lipoprotein cholesterol
LOPO	leave-one-participant-out
LXRα	liver X receptor-α
max-CWT	maximum carotid wall thickness
MUFA	monounsaturated fatty acid
OLS	ordinary least squares
OR	odds ratio
PLA	People’s Liberation Army
PUFA	polyunsaturated fatty acid
SCD1	stearoyl-CoA desaturase 1
SCD1-16	palmitoleic acid-to-palmitic acid ratio
SCD1-18	oleic acid-to-stearic acid ratio
SD	standard deviation
SFA	saturated fatty acid
Th17	T helper 17 cell
Treg	regulatory T cell
TyG	triglyceride–glucose index
WHR	waist-to-hip ratio
