# Supplementary material for: Dried Blood Spot Whole-Blood Fatty-Acid Profiles and Maximum Carotid Wall Thickness in Chinese Adults with Carotid Structural Involvement: A Dual-Route Compositional Cross-Sectional Study
Source: Nutrients. 2026 Oct 24;18(21):3495. doi: 10.3390/nu18213495 (PMC13765099; doi:10.3390/nu18213495)
Supplement: Supplementary file 1 [file nutrients-18-03495-s001.zip › Additional File/Additional_file_S2_STROBE_Checklist_R2.pdf]

STROBE Statement — Checklist of items to include in reports of cross-sectional studies

Manuscript: Dried Blood Spot Whole-Blood Fatty-Acid Profiles and Maximum Carotid Wall Thickness in Chinese Adults with Carotid Structural Involvement: A Dual-Route Compositional Cross-Sectional Study (Nutrients)

Locations are given by section/subsection number, table and figure as they appear in the manuscript. Supplementary Tables S1–S20 form Additional file S1; the participant flow diagram is Figure 1 in the main text; this checklist is Additional file S2.

Item	STROBE recommendation	Location in this manuscript
**Title and abstract**		
1	(a) Indicate the study's design with a commonly used term in the title or the abstract.<br>(b) Provide an informative and balanced summary of what was done and what was found.	(a) Title and Abstract (the design term "cross-sectional" appears in the Title and in Methods 2.1).<br>(b) Structured single-paragraph Abstract (Background–Conclusion).
**Introduction**		
2	Explain the scientific background and rationale for the investigation being reported.	Introduction, paragraphs 1–3 (background, prior evidence and the knowledge gap).
3	State specific objectives, including any prespecified hypotheses.	Introduction, final paragraph (three prespecified aims; no formal directional statistical hypotheses were registered; multiplicity controlled by the prespecified pooled Benjamini–Hochberg FDR, Statistical analysis).
**Methods**		
4	Present key elements of study design early in the paper.	Methods 2.1 (single-centre cross-sectional design stated at the outset).
5	Describe the setting, locations, and relevant dates, including periods of recruitment, exposure, follow-up and data collection.	Methods 2.1 (setting and study period, December 2025–August 2026) and Methods 2.2 (recruitment).
6	(a) Give the eligibility criteria and the sources and methods of participant selection.<br>(b) For cross-sectional studies, give details on ascertainment of outcome and exposure.	(a) Methods 2.2 (eligibility and recruitment; Figure 1).<br>(b) Methods 2.3 (DBS exposure ascertainment) and Methods 2.4 (carotid outcome ascertainment).
7	Clearly define all outcomes, exposures, predictors, potential confounders and effect modifiers; give diagnostic criteria where relevant.	Methods 2.4 (outcome max-CWT and ECST stenosis; severity cut-offs); Methods 2.3 (fatty-acid exposures); Methods 2.5 (Model 3 covariates); non-standard abbreviations in the Abbreviations table before Acknowledgments.
8	For each variable give sources of data and details of methods of assessment (measurement); describe comparability where more than one group.	Methods 2.3 (DBS GC-FID measurement), Methods 2.4 (standardised carotid ultrasound) and Methods 2.5 (routine biochemistry, ISO15189 laboratory); one group under a single protocol.
9	Describe any efforts to address potential sources of bias.	Methods 2.4 (one certified sonographer, prescribed bilateral protocol); Results 3.1 and Supplementary Table S4 (repeat-read reliability); Methods 2.6 (prespecified FDR families with pooled correction); Results 3.3 and Supplementary Tables S6–S7 (influence and leave-one-participant-out checks).
10	Explain how the study size was arrived at.	Methods 2.2 and Results 3.1 (consecutive recruitment; 328 registered to 230 analysed; Figure 1).
11	Explain how quantitative variables were handled in the analyses; if grouped, state categories and why.	Methods 2.6 (per-1-SD standardisation; ln(max-CWT)); Methods 2.4 and Results 3.1 (prespecified severity categories).
12	(a) Describe all statistical methods, including those used to control for	(a) Methods 2.6 (OLS with HC3 robust standard errors; Model 3 adjustment; Benjamini–Hochberg

	confounding.<br>(b) Describe any methods used to examine subgroups and interactions.<br>(c) Explain how missing data were addressed.<br>(d) Cross-sectional: if applicable, explain analytical methods accounting for sampling strategy.<br>(e) Describe any sensitivity analyses.	FDR).<br>(b) Not prespecified: no subgroup or formal interaction tests; conditional targeted and alternative-outcome models instead (Methods 2.6; Results 3.6–3.7; Supplementary Tables S3, S13, S16).<br>(c) Methods 2.6 and Results 3.1 (complete cases; no imputation, n = 230).<br>(d) Single-centre consecutive sample; sampling boundary discussed in the Discussion (generalisability).<br>(e) Methods 2.6; Results 3.3–3.7; Supplementary Tables S2–S3, S6–S20; Figures 3–7.
**Results**		
13	(a) Report numbers of individuals at each stage (potentially eligible, examined, confirmed eligible, included) and reasons for non-participation.<br>(b) Give reasons for non-participation at each stage.<br>(c) Consider use of a flow diagram.	(a)–(b) Methods 2.2 and Results 3.1 (numbers and reasons at each stage).<br>(c) Figure 1 (participant flow diagram).
14	(a) Give characteristics of study participants and information on missing data.<br>(b) Indicate number of participants in each exposure category.<br>(c) Cross-sectional: report summary measures where relevant.	(a) Table 1 and Supplementary Table S5 (characteristics); completeness in Methods 2.6 and Results 3.1<br>(b)–(c) Table 1 and Results 3.2 (severity categories and summary measures).
15	Cross-sectional: report numbers of outcome events or summary measures.	Table 1 (characteristics and outcome summaries), Table 2 (primary adjusted associations) and Table 3 (composition-aware results).
16	(a) Give unadjusted estimates and, if applicable, confounder-adjusted estimates with precision (e.g. 95% CI); state confounders and why.<br>(b) Report category boundaries when continuous variables were categorised.<br>(c) Translate estimates into meaningful units.	(a) Table 2 (adjusted β with 95% CI); Supplementary Table S2 (sequential Models 0–3); confounders in Methods 2.5<br>(b) Methods 2.4 (severity boundaries).<br>(c) Results 3.3 (per-1-SD contrasts expressed as percent and millimetre differences); Abstract.
17	Report other analyses done (e.g. subgroup, interaction, sensitivity).	Results 3.3–3.7; Table 3; Figures 3–7; Supplementary Tables S2–S3, S6–S20.
18	Summarise key results with reference to study objectives.	Discussion, opening paragraph (key results against the three aims); section 5 Conclusions.
**Discussion**		
19	Discuss limitations, including sources of potential bias, imprecision, and multiplicity; discuss direction and magnitude of any bias.	Discussion, strengths and limitations paragraphs (single-centre cross-sectional design, no paired-matrix DBS validation, multiplicity, and the direction of possible bias).
20	Give a cautious overall interpretation, considering objectives, limitations, multiplicity and similar studies.	Discussion (comparison with MESA, Ding 2020, Lázaro 2021 and related evidence) and section 5 Conclusions.
21	Discuss the generalisability (external validity) of the study results.	Discussion, limitations/generalisability paragraph (Chinese adults with metabolic-risk features and carotid structural involvement).
**Other information**		
22	Give the source of funding and the role of the funders.	Declarations — Funding (Grant No. 2025-1-Z-01); ethics approval and trial registration in Methods 2.1 and Declarations — Ethics Statement (ChiCTR2500113737; Ethics No. S2025-647-02).

Item 12(b) is marked not prespecified because no subgroup or formal interaction analysis was planned; the conditional targeted and alternative-outcome analyses are listed under Items 12(e) and 17. This is a cross-sectional study with no follow-up, so the follow-up part of Item 14(c) is covered by cross-sectional summary measures.
